# Mapping ongoing nutrition intervention trials in muscle, sarcopenia, and cachexia: a scoping review of future research

**DOI:** 10.1002/jcsm.12954

**Published:** 2022-03-17

**Authors:** Camila E. Orsso, Montserrat Montes‐Ibarra, Merran Findlay, Barbara S. van der Meij, Marian A. E. de van der Schueren, Francesco Landi, Alessandro Laviano, Carla M. Prado

**Affiliations:** ^1^ Human Nutrition Research Unit, Department of Agricultural, Food and Nutritional Science University of Alberta Edmonton Alberta Canada; ^2^ Cancer Services Royal Prince Alfred Hospital Camperdown New South Wales Australia; ^3^ Bond University Nutrition and Dietetics Research Group, Faculty of Health Sciences and Medicine Bond University Gold Coast Queensland Australia; ^4^ Department of Dietetics and Foodservices, Mater Health Services Mater Hospital South Brisbane Queensland Australia; ^5^ Department of Nutrition, Dietetics and Lifestyle, School of Allied Health HAN University of Applied Sciences Nijmegen The Netherlands; ^6^ Department of Human Nutrition and Health Wageningen University and Research Wageningen The Netherlands; ^7^ Department of Geriatrics, Neurosciences and Orthopaedics Catholic University of the Sacred Heart Rome Italy; ^8^ Geriatric Department Fondazione Policlinico Universitario ‘Agostino Gemelli’ IRCCS Rome Italy; ^9^ Department of Translational and Precision Medicine Sapienza University of Rome Rome Italy

**Keywords:** Clinical trials, Nutrition intervention, Muscle, Sarcopenia, Cachexia, Dietary intervention

## Abstract

Muscle loss alone, or in the context of sarcopenia or cachexia, is a prevalent condition and a predictor of negative outcomes in aging and disease. As adequate nutrition is essential for muscle maintenance, a growing number of studies has been conducted to explore the role of specific nutrients on muscle mass or function. Nonetheless, more research is needed to guide evidence‐based recommendations. This scoping review aimed to compile and document ongoing clinical trials investigating nutrition interventions as a strategy to prevent or treat low muscle mass or function (strength and physical performance), sarcopenia, or cachexia. ClinicalTrials.gov and the WHO International Clinical Trials Registry Platform were searched up to 21 April 2021 for planned and ongoing trials. Randomized controlled trials with ≥20 participants per arm were included based on intent to explore the effects of nutrition interventions on muscle‐related outcomes (i.e. muscle mass or strength, physical performance, or muscle synthesis rate) in both clinical and non‐clinical conditions (i.e. aging). Two reviewers independently screened records for eligibility, and a descriptive synthesis of trials characteristics was conducted. A total of 113 trials were included in the review. Most trials (69.0%) enroll adults with clinical conditions, such as cancer (19.5%), obesity and metabolic diseases (16.8%), and musculoskeletal diseases (10.7%). The effects of nutrition interventions on age‐related muscle loss are explored in 31% of trials. Although nutrition interventions of varied types were identified, food supplements alone (48.7%) or combined with dietary advice (11.5%) are most frequently reported. Protein (17.7%), amino acids (10.6%), and *β*‐hydroxy‐*β*‐methylbutyrate (HMB, 6.2%) are the top three food supplements' nutrients under investigation. Primary outcome of most trials (54.9%) consists of measures of muscle mass alone or in combination with muscle strength and/or performance (as either primary or secondary outcomes). Muscle strength and physical performance are primary outcomes of 38% and 31.9% of the trials, respectively. These measurements were obtained using a variety of techniques. Only a few trials evaluate muscle synthesis rate either as a primary or secondary outcome (5.3%). Several nutrition studies focusing on muscle, sarcopenia, and cachexia are underway and can inform future research in this area. Although many trials have similar type of interventions, methodological heterogeneity may challenge study comparisons, and future meta‐analyses aiming to provide evidence‐based recommendations. Upcoming research in this area may benefit from guidelines for the assessment of therapeutic effects of nutrition interventions.

## Introduction

Loss of muscle mass with or without loss of function (i.e. muscle strength or physical performance) can occur naturally with aging or in the context of acute and chronic diseases.^1^, ^2^ Although the prevalence of low muscle mass and sarcopenia (i.e. low muscle mass and function) may differ according to the diagnostic criteria used, recent pooled analyses revealed that these conditions are highly prevalent among community‐dwelling^3^ and hospitalized older adults^4^ as well as patients with lung diseases,^5^ liver cirrhosis,^6^ cancer,^7^ and other diseases.^8^, ^9^, ^10^ Patients may also lose muscle in the presence of obesity (i.e. sarcopenic obesity) or without changes in body weight.^11^, ^12^, ^13^ Low muscle mass is also a defining criterion of cachexia, which is a catabolic condition further characterized by severe weight loss with or without loss of fat mass and inflammation due to underlying diseases, with devastating consequences for patients.^14^, ^15^, ^16^

Given the mechanical, structural, and metabolic functions of skeletal muscle, a growing body of evidence indicates that low muscle mass is associated with adverse outcomes and increased healthcare costs.^17^, ^18^ For instance, having low muscle is a predictor of infection risk, length of hospital stay, readmission, hospital complications, reduced physical function, and mortality.^18^, ^19^, ^20^ Thus, effective management strategies that can prevent and treat muscle loss are necessary to optimize health outcomes.

Adequate supply of protein and energy are essential to maintain muscle mass and promote its synthesis.^21^ Several clinical trials have been conducted to investigate the role of nutrition interventions, including protein and other nutrients, in countering muscle loss. As summarized in a recent umbrella review of studies in older adults without acute or chronic diseases, long‐term interventions (≥24 weeks) combining protein supplementation and resistance training showed a positive effect on both muscle mass and strength^22^; increased muscle mass was also observed with leucine (in those with sarcopenia only) or *β*‐hydroxy‐*β*‐methylbutyrate (HMB) supplements as well as with concurrent creatine supplementation and resistance training.^22^ Notably, omega‐3 fatty acids are also a promising nutrient under consideration for improved muscle health in older adults and clinical conditions.^23^, ^24^ Furthermore, recent research has shown evidence of beneficial effects of HMB supplementation on muscle mass and strength in a variety of clinical conditions.^25^

Much research is needed to advance our understanding on the impact of nutrition intervention trials in muscle, sarcopenia, and cachexia. For example, given the paucity of studies reporting on post‐intervention follow‐up assessment, long‐term effectiveness remains to be established.^22^ Little is also known regarding the role of multi‐ingredient supplements and nutrition interventions across individuals and diseases states.^11^, ^22^, ^26^, ^27^ Findings are often underpowered to detect differences in muscle mass changes, and heterogeneity across studies (e.g. dose, frequency, and duration of interventions) preclude a comprehensive comparison of results.^22^, ^28^, ^29^, ^30^, ^31^, ^32^ Furthermore, the number of studies is relatively small (particularly in clinical populations), often lack measures of dietary intake and adherence and are limited by the methodological challenges to assess muscle parameters.^25^, ^31^, ^32^, ^33^, ^34^, ^35^, ^36^, ^37^, ^38^, ^39^

In view of these limitations, and the rapid growing interest in the field, we conducted a scoping review with the overall aim of documenting ongoing registered randomized clinical trials (RCTs) on nutrition interventions as a strategy to prevent or treat low muscle mass or function, sarcopenia, or cachexia, to help inform future research.^27^ Our specific objectives were to identify the types of nutritional interventions being explored, the range of diseases or conditions associated with low muscle mass or function, sarcopenia, or cachexia, as well as primary and secondary outcomes being studied. This scoping review may provide a basis for planning future studies, reducing duplication efforts, and advancing knowledge translation to improve patients' outcomes.

## Methods

### Search strategy and eligibility criteria

The search was conducted in two clinical trials registries, the ClinicalTrials.gov and the World Health Organization International Clinical Trials Registry Platform (WHO ICTRP), on 20 January 2021 and updated on 21 April 2021 for planned and ongoing RCTs. The WHO ICTRP is a search portal that allows a single point of access to several primary source registries globally.^40^ We used a combination of terms related to muscle, sarcopenia, cachexia, and nutrition interventions in standard search interfaces (Supporting Information, *Table* S1), given their greater sensitivity than advanced searches.^41^ We piloted and adapted the search string by deleting terms not adding results; terms related to exercise interventions were not included as it was not our aim to report studies on exercise interventions alone. Results were downloaded and exported into a spreadsheet (Microsoft Excel®), and duplicates removed using the ‘Remove Duplicates’ feature. Two reviewers independently screened trial details for the eligibility criteria (*Table* S2); disagreements were solved by a third reviewer. An additional search in the source registry (e.g. Clinical Trials Registry ‐ India, The Netherlands Trial Register) of each eligible trial was conducted (on 10 May 2021) to identify potential updates after the initial search date. If the latter occurred, modifications were recorded, and trials with status updated to being completed were excluded. On the same day, a search was carried out in PubMed, Google, and Google Scholar using the trial identification number to assess if eligible trials had recently published their findings as original manuscripts or conference abstracts; trials with full findings (but not preliminary results based on participant enrolment) were excluded. After reviewing the records, at least three attempts were made to contact corresponding authors (by email) to acquire missing information on population, study design, interventions, and outcomes. If authors failed to respond, only those trials not clearly reporting the type of nutrition intervention were excluded (*Table* S3).

### Data extraction and synthesis

Information on trial characteristics was primarily extracted from the source registries or through direct communications with corresponding authors. Trials with published protocols were also reviewed for relevant information if information was missing in the registry. Websites of nutritional supplement companies were consulted for details on supplement composition when needed. From these sources, we collected data on population characteristics, study design, interventional approach, primary and secondary outcomes, and other information (*Table* S4). Only those outcomes related to measures of muscle mass (i.e. quantity), muscle synthesis rate (assessed by muscle biopsy), and muscle function (i.e. muscle strength and physical performance) were retrieved. Search results were described using the Preferred Reporting Items for Systematic Reviews and Meta‐Analysis (PRISMA) flow diagram.^42^, ^43^ As reported below, trials were explored according to interventions and outcome measures as well as stratified by study population.

#### Interventions

Trials were sorted by type of nutrition intervention, food supplement, dietary advice, and multimodal interventions being provided to participants in the experimental arm. Nutrition interventions were categorized as food supplements, food modification, food products, fortified food products, and oral nutritional supplements (ONS) according to the definitions proposed by the European Society for Clinical Nutrition and Metabolism (ESPEN)^16^ (*Table* 1). The category ‘dietary advice’ was created to accommodate those trials providing participants with either dietary advice (by heath care providers, research staff, or self‐help sources) or counselling (by registered dietitians/nutritionists). We understand the limitations of using the terms ‘advice’ and ‘counselling’ interchangeably as they may differ in terms of the nature of intervention (e.g. nutrition education provided in‐person or using written information material vs. individualized person‐centred counselling), person delivering the intervention, length and type of follow up, and aims of intervention. Although we have attempted to extract these details when available (*Table* S5), we opted to not report nor evaluate these due to missing information in many trials.

**Table 1:** Terminology used in this scoping review to describe types of assessed nutrition interventions

Terminology	Definition	Examples
Dietary advice	Advice or counselling on healthy food choices provided by dietitians, health care providers, research staff, or self‐help sources.	Nutritional counselling, education sessions, support group, instructions provided via phone and written educational material.
Food product	Any food with nutrients and/or other substances that fulfils nutritional requirements.^16^	Dairy products (e.g. milk, cheese, and yogurt), meat, eggs, high‐carbohydrate snacks.
Food modification	Adjustments in the content of macronutrients and/or micronutrients in the diet according to an eating plan to achieve nutritional goals specific to conditions or disorders.^16^	High‐protein diet, Mediterranean diet, diet that meets individual energy needs, energy‐restricted diet, diet for diabetes.
Fortified food	Addition of nutrients to food products to increase energy or nutrient density.^16^	Chocolates enriched with leucine, food products enriched with protein.
Food supplement	Food products with concentrated source of nutrients (single or mixed) or other substances that are used to supplement normal diet. They are sold in several dose forms and are to be consumed in measured small quantities.^16^	Whey protein, creatine, vitamins, amino acid mixture, BCAA, pre−/probiotics, HMB, omega‐3 fatty acids, botanic dietary supplements.
Oral nutritional supplements	Energy and nutrient‐dense solutions prepared as drinks or added to drinks and foods to be consumed orally when diet alone is insufficient to meet daily nutritional requirements.^16^	High‐protein ONS, high‐energy ONS.
Specialized oral nutritional supplements	Nutrient specific ONS designed with anti‐inflammatory ingredients or amino acid metabolites.^44^	HMB‐enriched ONS, omega‐3 enriched ONS.

BCAA, branched‐chain amino acids; HMB, β‐hydroxy‐β‐methylbutyrate; ONS, oral nutritional supplement.

Food supplements were grouped based on the main nutrient component or derivatives/ingredients [e.g. proteins, amino acids, β‐hydroxy‐β‐methylbutyrate (HMB), creatine, botanicals, and vitamins]. As all proteins are composed of amino acids, we classified as ‘amino acid supplements’ only those interventions that described its amino acid composition [e.g. essential amino acids, branched‐chain amino acids (BCAA)]. Botanical supplements were defined as those food supplements made from plants, plant parts, or plant extract with the intent of supplementing the diet despite whether they met official definitions for dietary supplements within the country regulating the trial. Oral nutritional supplements (ONS) were classified based on the presence of specific nutrients (i.e. omega‐3 fatty acids) or ingredients (i.e. HMB) as specialized ONS^44^; those ONS not containing any of these specific nutrients or ingredients were classified simply as ‘ONS’ or ‘high‐protein ONS’. Due to missing information from one trial, we were unable to classify ‘Chinese medicine made diet’ within any of the proposed nutrition intervention categories; this trial was reported separately. For synthesis purposes, multimodal interventions (defined as nutritional interventions combined with other approaches targeting muscle mass) were classified into exercise (i.e. aerobic and resistance training and physical activity programmes), physical rehabilitation (i.e. physical therapy and electrical muscle stimulation), and drug therapy (i.e. testosterone). We also described whether patients are receiving treatment for their clinical conditions, such as surgery (i.e. solid organ transplant, cancer surgery, bariatric surgery, and orthopaedic surgery), drug therapy (i.e. chemotherapy and androgen deprivation therapy), or psychological therapy.

#### Outcome assessment

Trials were grouped according to the type of outcome measure (primary vs. secondary as reported in the registry), category of muscle‐related outcomes [i.e. muscle mass, muscle synthesis rate, and muscle function (i.e. muscle strength, and physical performance)], and assessment methods. For muscle mass assessment, trials were grouped based on the body composition technique [e.g. dual‐energy X‐ray absorptiometry (DXA), CT, and ultrasound] or anthropometric approach being employed. Due to lack of information in some studies, trials using bioimpedance techniques [e.g. bioimpedance electrical analysis (BIA) or bioimpedance spectroscopy] were hereby reported using the abbreviation ‘BIA’, although we understand that differences between techniques do exist, especially in terms of approaches used to estimate muscle mass.^45^ Given the diversity of approaches used to assess muscle strength, we grouped techniques into handgrip strength (HGS), one‐repetition maximum (1‐RM) test, and upper body strength or lower body strength (independently whether measures are obtained using isometric or isokinetic dynamometry). Likewise, physical performance tests were grouped into gait speed tests [e.g. 6 min walking test (6MWT), 6 m walk test, and 10 m walk test], short physical performance battery (SPPB), timed up and go (TUG), chair raise tests (e.g. chair stand, 30 s chair stand test, and sit to stand test), balance, stair climb, and VO_2max_. We considered a category of muscle‐related outcome as a ‘primary outcome’ for trials evaluating primary and secondary outcomes concurrently within the same category of muscle‐related outcome but using different assessment methods. Readers are referred to data reported in *Table* S4 if a different analysis is deemed necessary.

#### Study population

Trials were classified as including participants with clinical conditions (i.e. acute or chronic conditions) and non‐clinical conditions (i.e. aging with or without low muscle mass or function or sarcopenia). Trials enrolling participants with clinical conditions were further grouped based on the type of clinical conditions (e.g. cancer, obesity, chronic kidney disease, and musculoskeletal diseases). We also classified trials according to the presence of low muscle mass or function or sarcopenia (i.e. low muscle mass combined with low muscle function) in both clinical and non‐clinical conditions; cachexia was reported in clinical conditions as per trial definitions. The presence of weight loss, malnutrition, and frailty were described as these relate to muscle health.

## Results

A total of 2210 records were identified through the electronic searches (*Figure* *S1* *)*. Of these, 132 were deemed eligible and were assessed for study status updates and completeness of information. Fifteen records were excluded due to changes in study status. Although most corresponding authors responded to our inquiries (46 out of 72 emails sent), five records were excluded as we were unable to determine the type of nutrition intervention from the information provided (*Table* S3). A total of 112 records remained eligible and were therefore included. Two different studies were registered under the same trial identification number (*RBR‐9snttn*; http://ensaiosclinicos.gov.br/rg/RBR‐9snttn) and are therefore hereby treated as separate clinical trials. Thus, a total of 113 trials were analysed in this scoping review. For consistency, description of the trials is presented as ongoing, despite recruitment status reported in registries (e.g. recruiting, enrolling by invitation, and not yet recruiting).

### Overall characteristics of included trials

**Figure 1:**
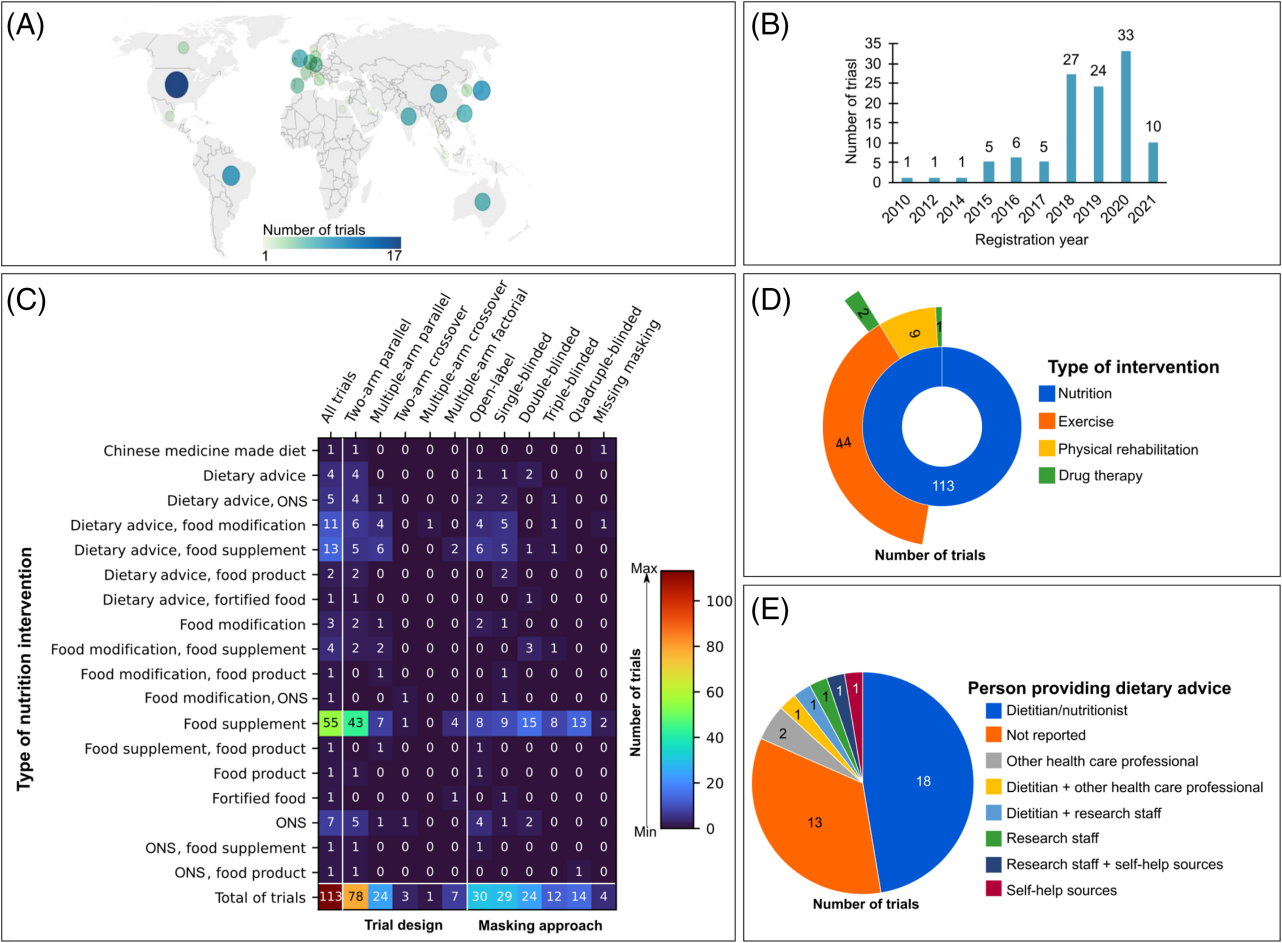
Graphical summary of the overall characteristics of ongoing randomized controlled trials investigating the effects of nutrition interventions on muscle mass or function, sarcopenia, or cachexia (*N* = 113). Numbers are absolute counts. (*A*) Illustration showing the distribution of trials according to country of registration. As depicted above, the USA is the leading country in the number of registered trials (15.0% of all trials); Brazil (8.0%) and Japan (8.0%) appears as the second leading countries. (*B*) Bar graph reporting number of trials per year of registration. (*C*) Heat map illustrating number of trials stratified by type of nutrition intervention, study design, and masking approaches. Colours within the heat map range from dark blue (least frequency) to dark red (most frequency); total numbers of trials can be found in the bottom row. (*D*) Sunburst chart showing the distribution of trials (in absolute counts) in which nutrition interventions (inner ring) are combined with one (middle ring) or ≥2 co‐interventions (outer rings; multimodal interventions). (*E*) Pie chart depicting how dietary advice is provided in 38 trials. Numbers placed inside of each piece of rings (in *D*) or pies (in *E*) correspond to the number of trials being studied. ONS, oral nutritional supplement.

Trials are being conducted in 27 countries, with many registered between 2018 and 2020 (*Figure* 1A and 1B). Relative to the total number of studies (*n* = 113), most trials (69.0%) enrol adults with clinical conditions, and the remaining (31.0%) enrol older adults with non‐clinical conditions. The presence of low muscle mass (5.3%) or function (2.7%) alone, sarcopenia (9.7%), and pre‐cachexia or cachexia (2.7%) are reported as inclusion criteria. About 69% of trials are randomizing participants to two intervention arms using a parallel assignment; of all trials, most are open‐label (26.5%), single‐blinded (25.7%), or double‐blinded (21.2%) (*Figure* 1C). Two trials report having a pragmatic design. The smallest trial includes 20 participants (cross‐over assignment), and the largest trial aims to enrol 3000 participants (three‐arm, parallel assignment). Almost 80% of trials enrol individuals of both sexes. The shortest and longest length of intervention are 5 days and up to 24 months (subject to change depending on adherence to the intervention), respectively. Follow‐up upon completion of the nutrition intervention ranges from 12 weeks to up to 24 months after the start of intervention. Multimodal interventions are reported in 47.8% of trials, with nutritional interventions being combined with one (46.0%) or two additional interventions (1.8%) (*Figure* 1D). Exercise or physical rehabilitation are provided in both experimental and control arms in 24.8% of trials (*Tables* S6 and S7).

**Figure 2:**
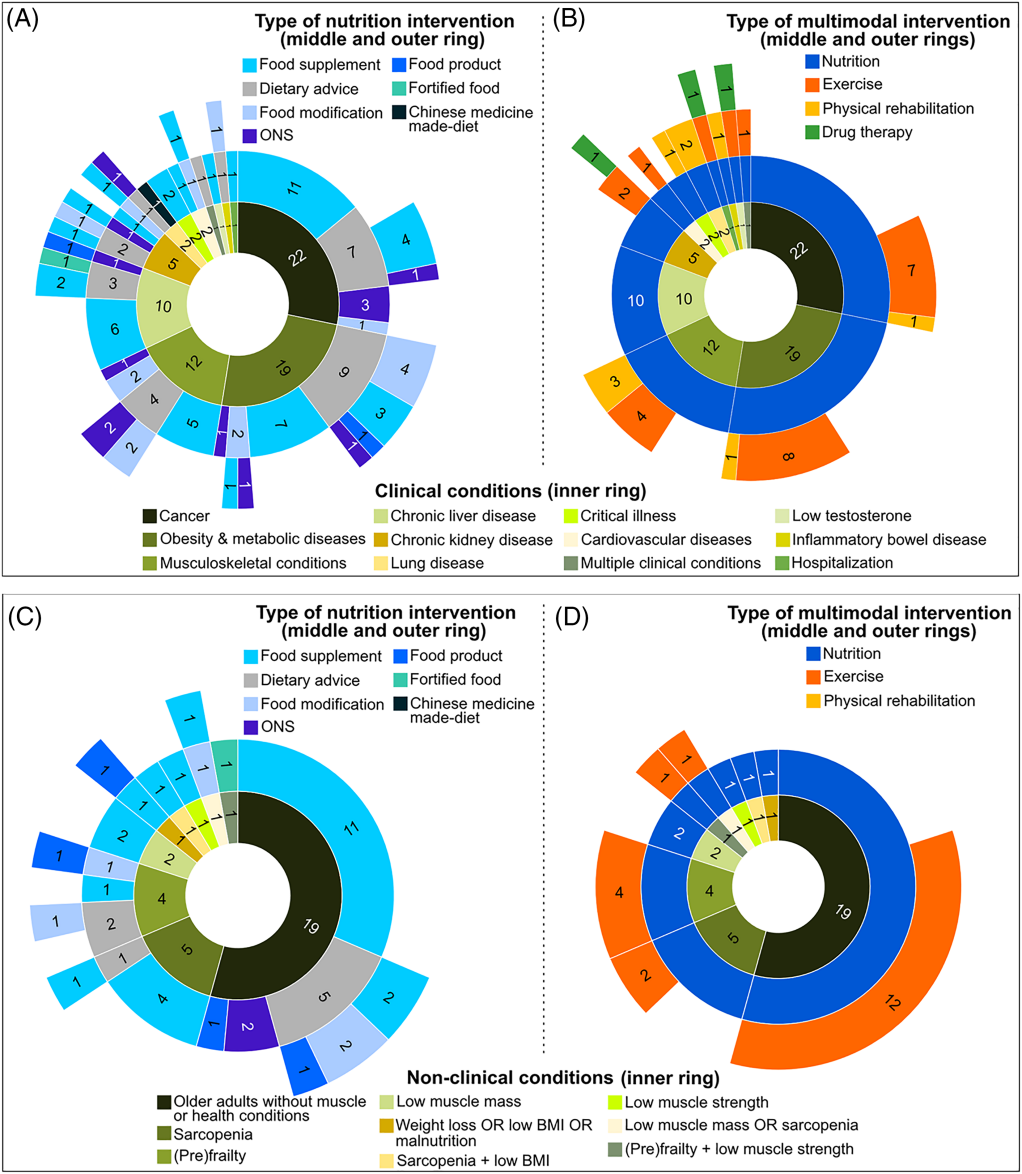
Sunburst charts depicting the types of nutrition and multimodal interventions under investigation in ongoing randomized clinical trials in (*A*, *B*) patients with clinical conditions (i.e. acute or chronic diseases; 78 out of 113 trials) and (*C*, *D*) older adults with non‐clinical conditions (with or without muscle‐related conditions; 35 out of 113 trials). While the inner rings represent categories of clinical and non‐clinical conditions, middle and outer rings describe the types of nutrition and multimodal interventions that relates to each condition. Numbers placed inside of each piece of rings correspond to the number of trials being studied in absolute counts. As an example of interpretation, in (*A*) of the 22 trials including patients with cancer, 11 provide patients with food supplements alone, 7 prescribe dietary advice (concurrent with food supplement in 4 trials or with ONS in 1 trial), 3 trials provide ONS alone, and 1 trial prescribe food modification alone. BMI, body mass index; ONS, oral nutritional supplement.

**Figure 3:**
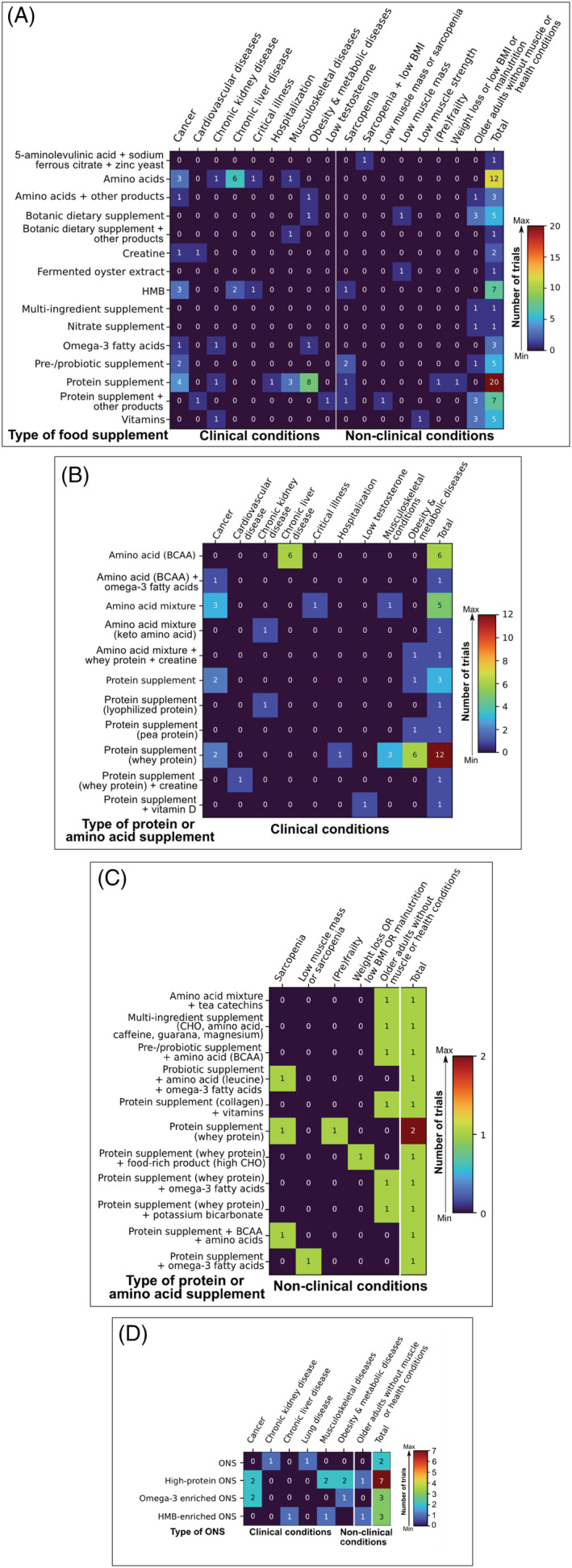
Heat map showing the distribution of ongoing randomized clinical trials studying the effects of food supplements (*n* = 74) and oral nutritional supplements (*n* = 15) on muscle mass or function, sarcopenia, or cachexia out of 113 trials included in this scoping review. (*A*) Types of food supplements under investigation in clinical (i.e. acute or chronic diseases; 49 out of 78 trials) and non‐clinical conditions (i.e. aging with or without muscle conditions; 25 out of 35 trials). (*B*) Composition of protein and amino acids supplements in clinical conditions. (*C*) Composition of protein and amino acids supplements in non‐clinical conditions. (*D*) Type of oral nutritional supplement in clinical and non‐clinical conditions. Colours within the heat map range from dark blue (least frequency) to dark red (most frequency). Value in each cell is absolute count, and the last column of each chart depicts total counts. Note that some clinical and non‐clinical conditions were omitted from the figure as they do not report nutrition interventions with food supplements or oral nutritional supplements. BCAA, branched‐chain amino acids; BMI, body mass index; Ca, calcium; CHO, carbohydrate; HMB, β‐hydroxy‐β‐methylbutyrate; ONS, oral nutritional supplements.

Several approaches are used as nutrition interventions (*Figure* 2). Most trials provide participants with food supplements alone (48.7%) or combined with dietary advice (11.5%). Of all trials, food supplements containing protein (17.7%), amino acids (10.6%), and HMB (6.2%) are the top three under investigation (*Figure* 3). Whey protein is the most reported protein type in food supplements (25.7% within food supplement trials; 16.8% of all trials), with doses ranging from 20 to 47.4 g/day or as necessary to set a specific intake level based on grams of protein per kilogram of body weight per day. Four trials are comparing different doses of protein across participants (*Table* S6 and S7). Amino acid supplements are provided either in the form of a mixture (19.8 mg/day to 46 g/day of essential amino acids) or BCAA (12–24 g/day). Participants receive food supplements containing HMB alone [3 g/day of calcium HMB (Ca‐HMB)] or combined with amino acids (3.0 g/day of Ca‐HMB, 14 g/day l‐arginine, and 14 g/day l‐glutamine). Oral nutritional supplements are provided in 13.3% of trials; of these, trials are studying the effects of ONS enriched with HMB (2.7%) or omega‐3 (2.7%) on muscle‐related outcomes. Refer to *Figure* 3 and *Tables* S6 and S7 for other supplements and dosages, respectively.

**Figure 4:**
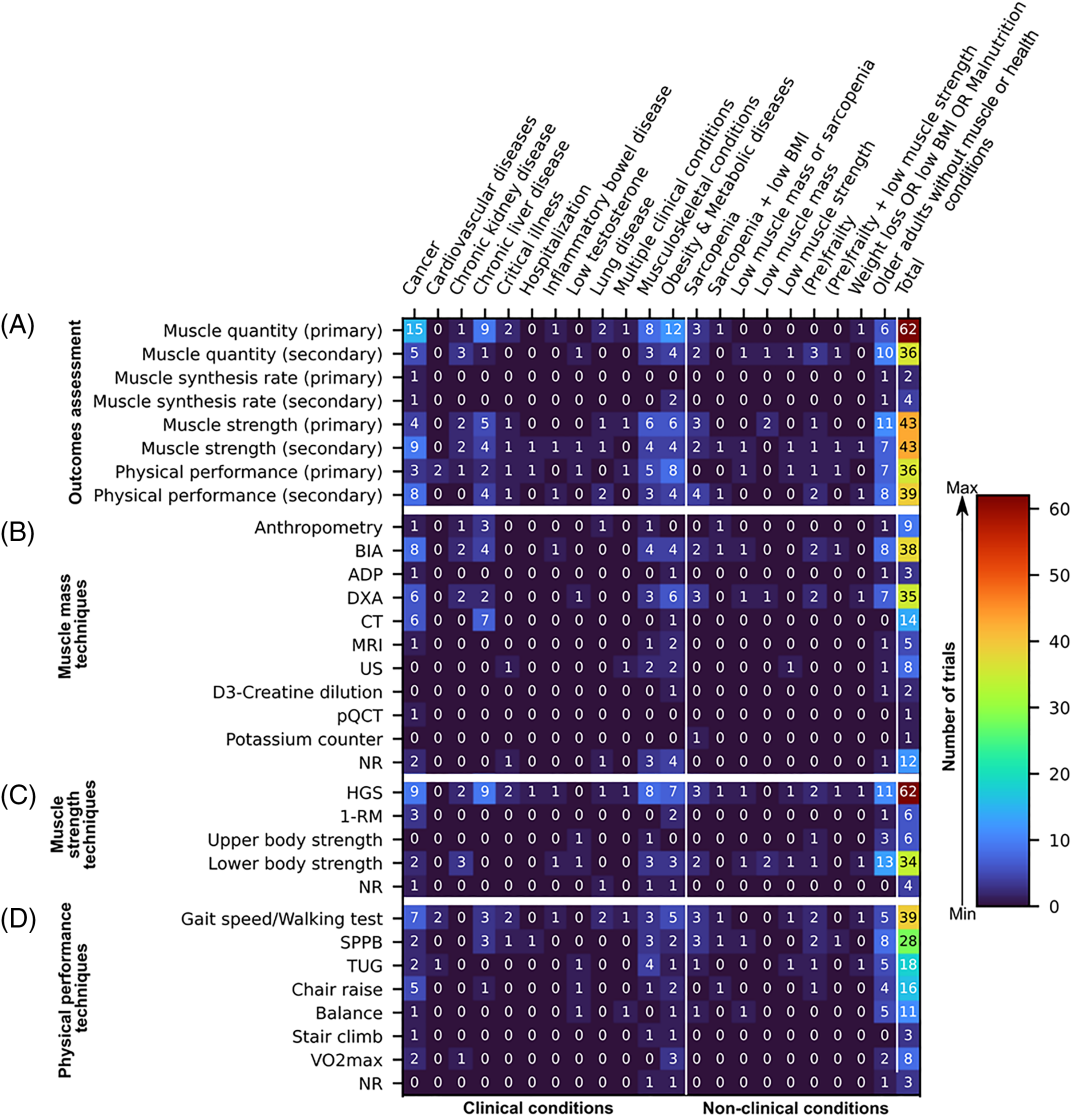
Heat map displaying outcome measures (*y*‐axis) of 113 ongoing randomized clinical trials studying the effects of nutrition interventions on muscle mass or function, sarcopenia, or cachexia. Trials are grouped by clinical and non‐clinical conditions (*x*‐axis). (*A*) Categories of muscle‐related outcomes (i.e. muscle quantity, muscle synthesis rate, muscle strength, and physical performance) stratified by types of outcomes (i.e. primary vs. secondary). (*B*) Anthropometric and body composition techniques being used to estimate outcomes related to muscle mass quantity. (*C*) Techniques being employed to evaluate muscle strength. (*D*) Methods commonly used to assess physical performance; note that different tests to evaluate gait speed and chair raise were grouped together for concision. Colours within the heat map range from dark blue (least frequency of trials) to dark red (most frequency of trials). Value in each cell is absolute count, and the last column of each figure panel depicts total counts. Note that some trials reported one or more concurrent primary or secondary outcomes. 1‐RM, one‐repetition maximum; ADP, air‐displacement plethysmography; BIA, bioelectrical impedance analysis; BMI, body mass index; CT, computed tomography; DXA, dual‐energy x‐ray absorptiometry; HGS, handgrip strength; MRI, magnetic resonance imaging; NR, not reported; pQCT, peripheral quantitative computed tomography; SPPB, Short Physical Performance Battery; TUG, timed up and go; US, ultrasound.

Most trials (54.0%) include measures of muscle mass (either as primary or secondary outcome) in combination with both muscle strength and physical performance (either as primary or secondary outcomes) (*Figure* 4). Measures of muscle mass are obtained as primary outcomes in 54.9% of trials; of these trials (*N* = 62), 14.5% evaluate muscle mass as the sole primary outcome, 25.8% combine measures of muscle mass with muscle strength and physical performance as primary outcomes concurrently, and 9.7% and 3.2% combine measures of muscle mass with muscle strength or physical performance as a co‐primary outcome, respectively. Although BIA (33.6%), DXA (31.0%), and CT (12.4%) appear as the top three body composition techniques, muscle mass is also being estimated by anthropometry (8.0%), ultrasound (7.1%), magnetic resonance imaging (4.4%), air‐displacement plethysmography (2.7%), D_3_‐creatine dilution (1.8%), peripheral quantitative computed tomography (0.9%), and potassium counter (0.9%). Muscle strength is measured as a primary (37.7%; of these, 11.6% assess muscle strength as the sole primary outcome) or secondary outcome (37.7%), and the HGS test is the most common approach (54.9%) chosen by these trials followed by measures of lower body strength (30.1%). Physical performance is evaluated as a primary (31.9%; of these, 13.9% assess physical performance as the sole outcome) or secondary outcome (34.5%) using gait speed (34.5%), SPPB (24.8%), TUG (15.9%), and other techniques. A small number of studies evaluate muscle synthesis rate either as a primary (1.8%; only one study assesses muscle synthesis rate as the sole outcome measure) or secondary outcome (3.5%).

### Trials in clinical conditions

#### Cancer

**Figure 5:**
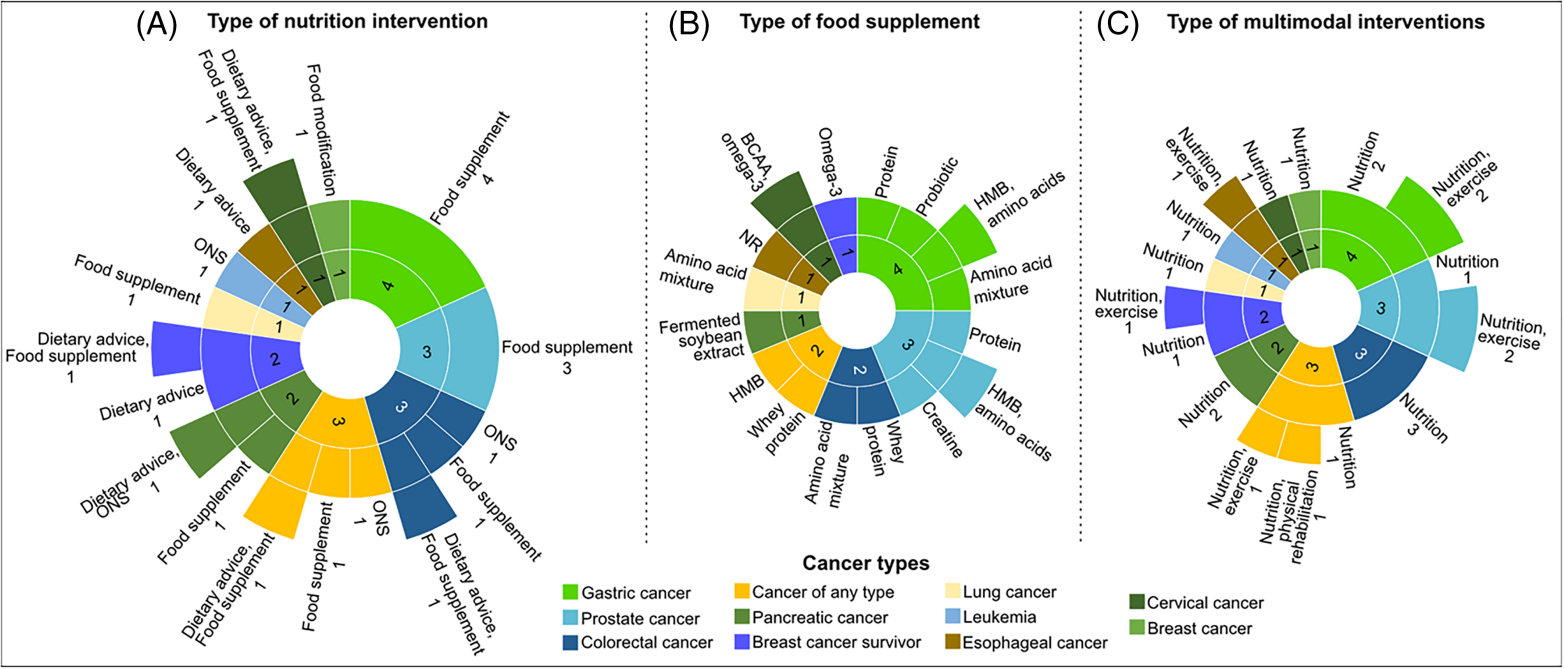
Sunburst charts illustrating the characteristics of interventions and distribution of trials across cancer types (inner rings, *n* = 22). Labels are placed outside of rings to indicate the number of trials and types of (A) nutrition interventions, (B) food supplements, and (C) multimodal interventions. Values are absolute counts. Nutrition interventions are given before cancer surgery in four trials, after cancer surgery in two trials, and both pre‐ and postoperatively in two trials. Patients included in eight trials are undergoing chemotherapy or radiotherapy while receiving the nutrition intervention. All patients with prostate cancer are receiving androgen deprivation therapy. One trial includes patients who are undergoing either curative or palliative cancer treatment, although therapy type was not specified. BCAA, branched‐chain amino acids; NR, not reported; ONS, oral nutritional supplement.

Twenty‐two (19.5%) trials in patients with cancer (aged ≥18 years) were identified. Studies enrol 40 to 312 patients with a variety of cancer types (*Figure* 5). Seven studies include patients with concurrent pre‐cachexia or cachexia (2.7% of all studies), malnutrition (1.8%), weight loss in the prior month ≤10% (0.9%), or chemotherapy‐induced peripheral neuropathy (0.9%). Only one trial described the criteria to define cachexia at registration, which was defined as weight loss alone (>2%; pre‐cachexia) or weight loss combined with low muscle mass (appendicular skeletal mass index by DXA ≤ 7.23 kg/m^2^ in men and ≤5.67 kg/m^2^ in women).^46^

Most trials provide participants with food supplements alone or with concurrent dietary advice, with the length of nutrition interventions ranging from 3 weeks to 6 months. Dietary advice differed between trials as reported in *Table* S5; however, this information was not systematically collected as discussed in the Methods section. Food supplements include proteins, amino acids, HMB alone or combined with amino acids, omega‐3 fatty acids, creatine monohydrate, and probiotics (*Figure* 3). Trials in patients with pre‐cachexia or cachexia prescribe food supplements composed of whey protein, fermented soybean extract, or a combination of BCAA and omega‐3 fatty acids supplements in addition to dietary advice with the goal of achieving a high‐protein diet. Additionally, ONS [either high‐protein (in two trials) or enriched with omega‐3 fatty acids (in two trials)], dietary advice alone, and a calorie‐restricted diet are prescribed to participants without cancer cachexia. A summary of nutrition interventions and specific targeted nutrients stratified by cancer type is provided in *Figure* 5, and dosages are described in *Table* S6. Multimodal interventions include nutrition interventions in combination with exercise or physical rehabilitation in eight trials (*Figures* 2B and 5C).

Most studies assess muscle mass as a primary outcome followed by measures of muscle strength and physical performance as secondary outcomes (*Figure* 4). Techniques most used to estimate muscle mass are BIA, DXA, and CT. Two studies assess muscle protein synthesis rate. Handgrip strength and gait speed appear as common approaches to evaluate muscle strength and physical performance, respectively.

#### Obesity and metabolic diseases

**Figure 6:**
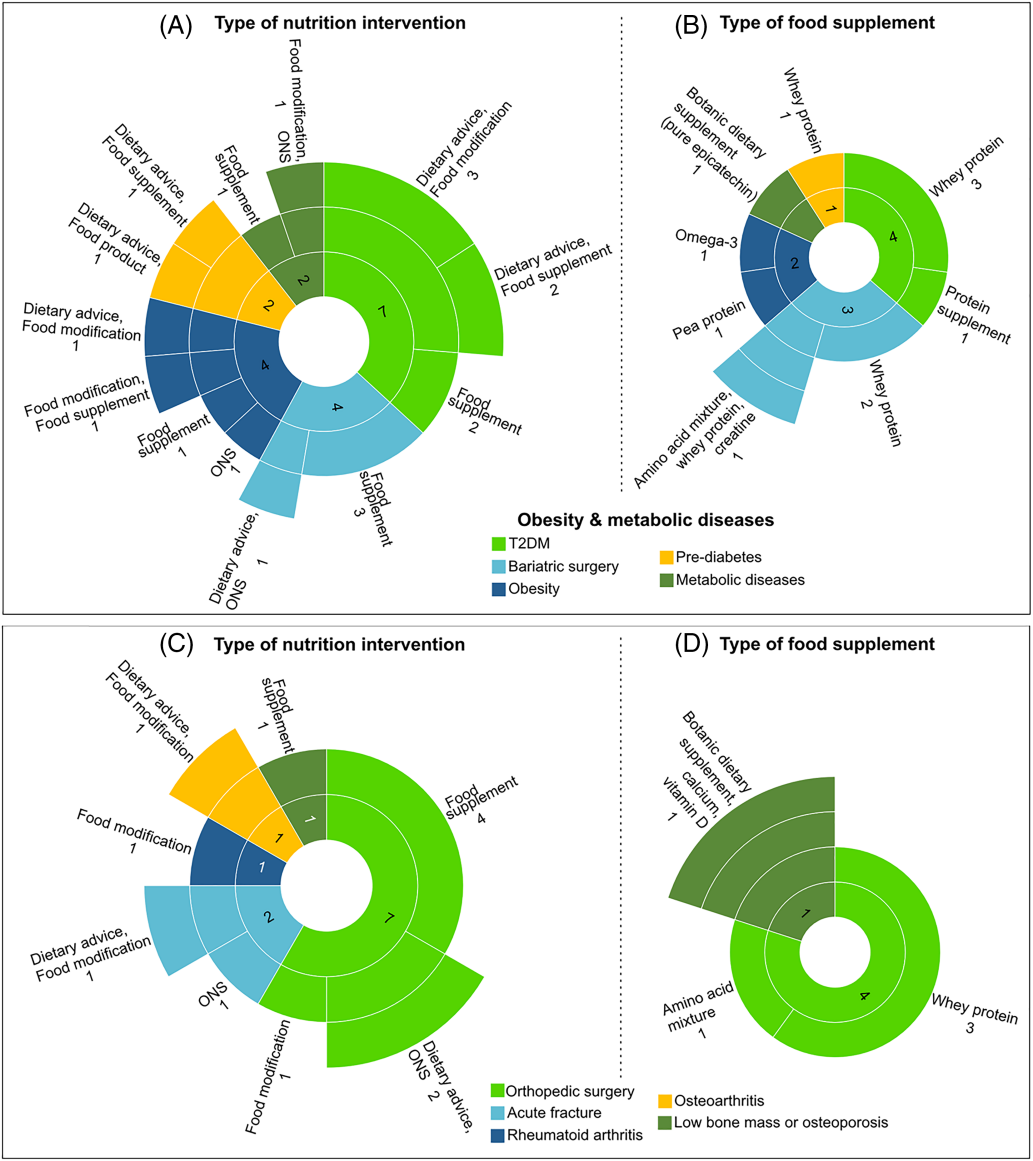
Sunburst charts showing characteristics of interventions and distribution of trials across patients with obesity and metabolic diseases (*A*, *B*; *n* = 19 trials) as well as musculoskeletal conditions (*C*, *D*; *n* = 12). While the inner rings represent classes of conditions, middle and outer rings describe the types of nutrition interventions (*A*, *C*) and food supplements (*B*, *D*) that relates to each condition. Labels are placed outside of rings to describe these information and number of trials (in absolute counts). Numbers placed inside of each innermost piece of rings correspond to the total number of trials being studied in each condition. HMB, β‐hydroxy‐β‐methylbutyrate; ONS, oral nutritional supplement; T2DM, type 2 diabetes mellitus.

Eight trials (7.1%) include individuals with obesity aged 18–80 years (*Figure* 6A). Of these, four trials investigate the effects of nutrition support after bariatric surgery. Target sample sizes range from 40 to 100 participants, and length of nutrition intervention from 8 weeks to 6 months. Nutrition interventions include dietary advice alone or combined with food supplements (mostly whey protein) or high‐protein ONS (*Figure* 6A and 6B; *Table* S6). Patients being enrolled in two trials also participate in an exercise programme in addition to the nutrition intervention (*Figure* 2B).

Nine trials (8.0%) include patients with either pre‐diabetes or type 2 diabetes (*Figure* 6A). Target sample sizes range from 40 to 1000 participants, and length of nutrition intervention from 10 to 52 weeks. Two studies enrol patients with type 2 diabetes and sarcopenia concurrently. Of these, only one study described the diagnostic criteria for sarcopenia, which includes low HGS (or gait speed) and low muscle mass (cut‐off points were not disclosed). Nutrition interventions comprise protein supplements, dietary advice, or high‐protein food products. A multimodal intervention with exercise or physical rehabilitation is provided in seven of these trials. Two studies in patients with metabolic diseases were also identified (*Figure* 6A).

Most trials in obesity and metabolic diseases evaluate muscle mass, muscle strength, physical performance or a combination of them as primary outcomes (*Figure* 4). Two clinical trials assess muscle synthesis rate as a secondary outcome. Body composition techniques mostly reported are DXA and BIA. Handgrip strength and gait speed are the methods of choice in most trials to assess muscle function.

#### Musculoskeletal conditions

Twelve nutrition trials (10.7%) in patients with diverse musculoskeletal conditions were identified (*Figure* 6C), including individuals who either have had or are awaiting orthopaedic surgery, older adults temporarily immobilized and recovering from an acute fracture, and adults with osteoarthritis, rheumatoid arthritis, or low bone mass or osteoporosis. Target sample sizes range from 40 to 400 participants. Studies provide participants with food supplements, high‐protein ONS or ONS enriched with HMB, or food modification alone or combined with dietary advice (*Figures* 2A and 3). *Figure* 6C and 6D provides a summary of nutrition interventions and food supplements stratified by musculoskeletal conditions. Most trials collect measures of muscle mass, muscle strength, physical performance, or a combination of them as primary outcomes using several techniques (*Figure* 4).

#### Chronic liver diseases

Ten trials (8.9%) aim to enrol 40 to 150 patients with liver cirrhosis (*Figure* 2A). Five are being conducted in patients with low muscle mass (CT‐based measures at L3), low muscle strength (by HGS), or sarcopenia (by CT and HGS). Most trials provide BCAA supplements alone or combined with dietary advice (*Figures* 2A and 3B; *Table* S6). Trials also evaluate the effects of HMB alone or ONS enriched with HMB in combination with a low‐glycaemic index carbohydrate as a late evening snack (*Figure* 3; *Table* S6). Two studies, registered under the same trial identification number (*RBR‐9snttn*; http://ensaiosclinicos.gov.br/rg/RBR‐9snttn), enrol patients on the waiting list for liver transplantation. Patients in these studies receive a meal plan and dietary advice to achieve a high‐protein diet combined with either a leucine‐enriched food (7.5 g/day leucine; study 1) or a HMB supplement (study 2). No multimodal interventions were noted. Skeletal muscle mass obtained by CT scans is assessed as a primary outcome in most trials (*Figure* 4). Handgrip strength is evaluated as the sole measure of muscle strength either as a primary or secondary outcome. Physical performance is assessed mostly as a secondary outcome using either SPPB or 6MWT.

#### Chronic kidney diseases

Five trials (4.4%) include patients with chronic kidney diseases (*Figure* 2A). In two kidney transplant studies, patients receive either a preoperative multimodal intervention for 8 weeks (i.e. dietary advice combined with protein supplement, exercise programme, and psychological advice) or dietary advice alone for 12 months with the goal to maintain ideal body weight after surgery. Two additional trials investigate the effects of food supplement alone (vitamin D) or in the context of a multimodal intervention (omega‐3 fatty acids supplement plus ONS, exercise, and testosterone therapy) on clinical outcomes during haemodialysis treatment (refer to *Table* S6 for dosages). Furthermore, one trial uses a low‐protein diet (0.6 g/kg BW/day) combined with keto‐amino acids supplements for 12 months. Muscle mass is estimated by BIA and DXA in most trials as a secondary outcome (*Figure* 4). Trials evaluate muscle strength either as primary or secondary outcome by lower body strength tests and HGS. Only one study measures VO_2max_ as a primary outcome for physical performance.

#### Lung diseases

Two trials (1.8%) enrol patients with chronic obstructive pulmonary disease (COPD) (*Figure* 2A). One trial includes 90 participants with malnutrition, and patients receive ONS and dietary advice. In another trial, 122 participants with malnutrition and low fat‐free mass are given a Chinese medicine made‐diet. Both trials have a length of intervention of 12 weeks, and patients receive physical rehabilitation in addition to nutrition support. Outcome measures include estimation of muscle mass by anthropometry, muscle strength (HGS), and physical performance (6MWT) (*Figure* 4).

#### Critical illness

We identified two parallel randomized studies (1.8%) in patients with critical illness requiring mechanical ventilation (*Figure* 2A, *Table* S6); no information on critical care setting was provided. Target sample sizes range from 60 to 68 patients. In one study, patients receive a HMB supplement (as a single nutrient) for up to 28 days via enteral tube feeding or orally depending on their ability to eat. In another study, patients receive a multimodal intervention consisting of protein supplement in combination with physical rehabilitation and neuromuscular electric stimulation for 14 days. Primary and secondary outcomes include measures of muscle mass (by ultrasound), muscle strength (by HGS), and physical performance (6MWT and SPPB) (*Figure* 4).

#### Cardiovascular diseases

Two nutrition trials (1.8%) in adults with cardiovascular diseases are being conducted (*Figure* 2A, *Table* S6). In one study, 60 post‐stroke patients receive whey protein and a creatine supplement for 7 days. In the other study, 40 patients with stable peripheral arterial disease receive creatine supplement for 4 weeks in addition to muscle stretching. Primary outcome includes only measures of physical performance by either the 6MWT or TUG (*Figure* 4).

#### Multiple clinical conditions

One trial (0.9%) investigates the effects of dietary advice combined with resistance exercise in 320 patients with stroke, osteoporosis, chronic kidney diseases, or cancer of any type (*Figure* 2A and 2B). The 3 month intervention includes muscle mass, muscle strength, and physical performance as concurrent primary outcomes. It is unclear whether authors plan to conduct separate analysis by disease group.

#### Low testosterone

We also identified one trial (0.9%) including 196 older men with low serum testosterone (<10 nmol/L) (*Figure* 2A and 2B). Participants in the experimental arm are given protein supplement and vitamin D after every exercise session for 16 weeks. They also receive testosterone injection. Primary outcome assessment includes measures of physical performance, and secondary outcome assessment includes muscle mass and muscle strength (*Figure* 4).

#### Inflammatory bowel disease

One trial (0.9%) in patients with Crohn's disease and ulcerative colitis was identified (*Figure* 2A). The trial has a three‐arm parallel design and includes 75 patients of both sexes. Dietary advice targets a high‐protein diet (1.5 g/kg BW/day) combined with whole‐body electromyostimulation for 12 weeks. Primary outcome includes estimates of muscle mass (by BIA), and secondary outcomes include muscle strength (HGS and lower limb strength), and physical performance (6MWT) (*Figure* 4).

#### Hospitalized patients

In one trial (0.9%), 80 hospitalized older adults receive whey protein supplement in combination with testosterone injection for 30 days (*Figure* 2A and 2B; *Table* S6). Physical performance is assessed by the SPPB test as a primary outcome and muscle mass by HGS as a secondary outcome.

### Trials in non‐clinical conditions

#### Older adults without muscle or health conditions

Nineteen trials (16.8%) are recruiting 20 to 3000 middle‐aged and older adults (≥40 years old) without co‐morbidities, low muscle mass or function, sarcopenia, or cachexia (*Figure* 2C). Participants receive food supplements, high‐protein or HMB‐enriched ONS, or high‐protein food products. Most trials provide botanicals, protein, and vitamin supplements; a description of food supplements and dosage are provided in *Figure* 3 and *Table* S7. Dietary advice is provided alone or integrated to other nutrition interventions aiming to either increase protein intake or promote a Mediterranean eating pattern. Nutrition interventions are accompanied by exercise or physical activity programmes in 12 trials (*Figure* 2D). Length of nutrition intervention range between 5 days (acute bed rest intervention) and 12 months. One trial plan to extend the nutrition intervention up to 24 months if adherence to the programme is high. Most trials assess muscle mass as a secondary outcome using BIA and DXA (*Figure* 4). Muscle strength is evaluated mainly as a primary outcome, using HGS or lower body strength. Researchers also assess physical performance either as a primary or secondary outcome; most trials use the SPPB test.

#### Older adults with low muscle mass or function, or sarcopenia

Ten trials (8.9%) enrol between 52 to 200 older adults with low muscle mass or function, or sarcopenia (*Figure* 2C). Sarcopenia is defined by different diagnostic criteria [i.e. Asian Working Group for Sarcopenia,^47^, ^48^ European Working Group on Sarcopenia in Older People (EWGSOP2),^2^ or low muscle mass combined with low muscular strength (HGS) or physical performance (gait speed)^49^], although some trials used a combination of definitions. Most trials use food supplements of varied types (*Figure* 3, *Table* S7). In one trial, dietary advice is provided in addition to HMB supplementation. In another trial, participants receive one serving of a high‐protein ONS per day. These interventions last between 60 days and 24 weeks, and three trials also include an exercise programme. Measures of muscle mass and strength are obtained in most trials either as primary or secondary outcomes (*Figure* 4). Trials preferentially use BIA and DXA to estimate muscle mass, and HGS or lower body tests to evaluate muscle strength. Physical performance is being evaluated mainly as a secondary outcome using gait speed tests or SPPB.

#### Older adults with pre‐frailty or frailty

Five trials (4.4%) include between 150 and 1000 older adults with pre‐frailty or frailty, as defined by the Fried criteria,^50^ Rockwood Clinical Frailty Scale,^51^ or the Survey of Health, Aging and Retirement in Europe (SHARE)‐FI75+.^52^ Participants receive protein supplement, high‐protein food products, or dietary advice on how to achieve a high‐protein diet or healthy eating according to nutrition status (*Figure* 2C). One of these trials include individuals with both pre‐frailty and low muscular strength (assessed by HGS). All five trials integrate nutrition with exercise interventions, and they last between 12 weeks to 12 months (*Figure* 2D). Measures of muscle mass are obtained solely as secondary outcomes using BIA or DXA (*Figure* 4). Muscular strength and physical performance are assessed either as primary or secondary outcomes mainly using HGS and SPPB, respectively.

#### Older adults with malnutrition

We identified one trial (0.9%) enrolling 93 institutionalized older adults at risk of malnutrition or moderate malnutrition. Participants receive a whey protein at lunch and a high‐carbohydrate food product after dinner for 90 days (*Table* S7). Primary outcome of interest includes measures of muscle mass by DXA, and secondary outcomes are muscle strength (by HGS and lower body strength test) and physical performance (using the 6MWT and TUG tests) (*Figure* 4).

## Discussion

This scoping review identified ongoing registered clinical trials focusing on nutrition interventions as a strategy to prevent or treat low muscle mass or function, sarcopenia, or cachexia in adults with clinical and non‐clinical conditions. Despite the diversity of intervention approaches, our results indicate that food supplements and ONS composed of protein, amino acids, HMB, or omega‐3 fatty acids are of particular interest by researchers in the field given their potential for anabolic effects.^53^, ^54^ Although these are not novel nutrition interventions, as highlighted by a number of systematic reviews,^22^, ^28^, ^29^, ^30^, ^31^, ^32^, ^33^, ^34^, ^35^, ^36^ further research is needed to understand their impact on muscle mass and function.

One major question that remains elusive is the optimal dose to promote muscle protein synthesis in aging and clinical conditions, although a higher protein intake compared to needs of healthy young adults (except in severe chronic kidney disease) has been recommended.^21^, ^55^, ^56^, ^57^, ^58^ We found a wide range of dosage regimens under investigation in these trials, and only a few of them compare different protein doses within the same study. Moreover, controversies exist regarding whether larger protein doses (30 to 40 g) per meal should be recommended to optimize muscle anabolism.^33^, ^59^ Testing the impact of different protein doses (per day and meal) and distinct amino acid composition (e.g. high in essential amino acids) on muscle‐related outcomes is therefore recommended in future studies, as protein bioavailability is determined by many factors^11^, ^60^, ^61^, ^62^ and ongoing trials are not sufficiently addressing these questions.

Multimodal interventions may also enhance the effects of nutrition interventions, as shown for older adults,^22^, ^28^, ^33^, ^63^ but with limited evidence in clinical populations.^38^, ^64^, ^65^ We hereby demonstrate that a number of ongoing trials combine nutrition interventions with other synergistic strategies such as exercise programmes and drug therapies (i.e. testosterone). Although multimodal therapies may target a set of pathways related to muscle anabolic and/or catabolic response, we found that many multimodal trials allocate exercise interventions to both experimental and control arms (i.e. a ‘standard of care’ or ‘no intervention’ arm is absent). Ideally, the use of four arms is recommended to understand whether and how synergistic therapies lead to additional benefits to nutrition interventions,^31^ although our findings indicate that ongoing studies are not following this approach. A ‘standard of care’ arm is preferred over ‘no intervention’ because of ethical issues related to depriving appropriate treatment for those in need.^66^

The effectiveness of nutrition interventions to prevent or treat sarcopenic obesity is another timely research topic not being investigated by ongoing clinical trials.^67^, ^68^ It is possible that individuals with sarcopenic obesity respond differently to nutrition interventions due to anabolic resistance associated with obesity and lower baseline muscle mass and function, compared with individuals with sarcopenia or obesity alone.^59^ The recently proposed consensus definition of sarcopenic obesity may facilitate enrollment of a more representative sample of patients, potentially reducing the heterogeneity related to eligibility criteria in a future meta‐analysis.^69^, ^70^

Previous systematic reviews evaluating the effects of nutrition interventions on muscle health have also identified the absence of baseline protein and caloric intakes as well as reduced patients' adherence to the intervention protocol as potential sources of bias.^25^, ^32^, ^33^, ^34^, ^35^, ^36^, ^37^, ^71^ Although it was not our objective to collect such information, we noticed that several trials did not report measures of adherence (data not shown). As meeting energy and protein requirements may be challenging to some individuals, adherence to prescribed interventions should be considered and evaluated in all RCTs to inform whether intervention effects are in fact measurable at study completion.^39^ Trials may also provide participants with education and awareness on the importance of nutritional strategies to prevent muscle loss. Our findings highlight the common inclusion of dietary advice concurrent to nutrition intervention. In fact, nutritional counselling has been considered the first line for prevention and treatment of malnutrition, a risk factor for muscle loss, as it supports personalized nutritional strategies and improved health outcomes.^57^, ^58^, ^72^

One may argue that the study design most suitable to single nutrient trials may be the placebo‐controlled, double‐blinded, parallel arm as recommended in drug trials.^73^ This design is common among food supplement trials but may be challenging to be achieved in other nutrition or multimodal interventions, especially in studies providing dietary advice or counselling, food substitutes (e.g. high‐protein food products), or exercise where blinding participants is difficult, if not impossible. One approach to avoid bias would be to have outcome measures assessed by blinded researchers.^74^

Nevertheless, pragmatic and adaptive clinical trials may have advantages over traditional RCTs, with potential to shape the future landscape of nutrition research and reduce the time gap between knowledge generation and application in clinical practice.^75^, ^76^, ^77^ For instance, this study design allows for the tailoring of nutrition interventions toward individuals' nutritional needs by either embedding the intervention within routine care or testing multi‐arm trials of distinct treatments (including different doses), with results of interim analyses determining whether treatment arms can be included or dropped.^75^, ^78^, ^79^, ^80^ Moreover, data on healthcare resource utilization can be obtained from patients' electronic medical records and used toward cost‐effectiveness analysis, reducing economic burden in healthcare system.^81^, ^82^ Of the studies included here, only two use a pragmatic design and five will perform a cost‐effectiveness analysis, emphasizing the need for future trials to incorporate these approaches.

Regarding outcome assessment, our findings show that BIA and DXA are common techniques used to estimate muscle mass; notably, they provide double indirect and indirect measures of body composition, respectively.^83^, ^84^ Handgrip strength is being assessed in most trials; however, it may not be sensitive enough to detect longitudinal changes in muscle function,^85^ and other potential approaches including lower body strength, gait speed, SPPB, and TUG may be used instead. Alongside with measures of physical function, and in line with regulatory agencies expectations for drug trials, inclusion of outcomes related to quality of life should also be considered in future studies.^86^

We also noticed that several trials include one or more co‐primary outcome simultaneously. As not all trials clearly stated their research objectives, we could not determine if the reporting of multiple primary outcomes was truly intended or whether these trials will adjust the statistical analysis. Multiple primary outcomes are in fact not recommended for future nutrition intervention trials if adjustments are not considered, as they may inflate type I error rate precluding a greater probability of finding at least one false significant result.^87^, ^88^, ^89^ This issue is particularly relevant in studies where intervention success is defined by a significant effect observed in at least one of several outcomes.^89^

Although we have made a great effort to contact corresponding authors for clarification, not all replied. As such, we were unable to summarize details on food supplement or ONS type, composition, dosage, goals, person providing dietary advice, and outcome assessment for all trials. Furthermore, we were unable to assess whether trials provide nutrition interventions under ideal conditions (e.g. controlled‐feeding trial) or control background diet and total energy intake.^90^, ^91^ Another limitation is that we could not control for changes in trial status and other information provided, given the nature of clinical trial registries where investigators can update their studies at any moment. Despite that, we manually verified extracted data from each trial immediately before conducting the data synthesis. Lastly, quality of life is an important patient‐centred outcome to be included in nutrition trials, and not evaluating it as an outcome of interest in this scoping review is an additional limitation. We observed that 58 (51.3%) studies mentioned assessment of quality of life; however, this number may not be an accurate representation as there may be other ongoing clinical trials that are assessing the effects of nutrition interventions on quality of life but not on muscle parameters and were, therefore, not captured in our search.

**Figure 7:**
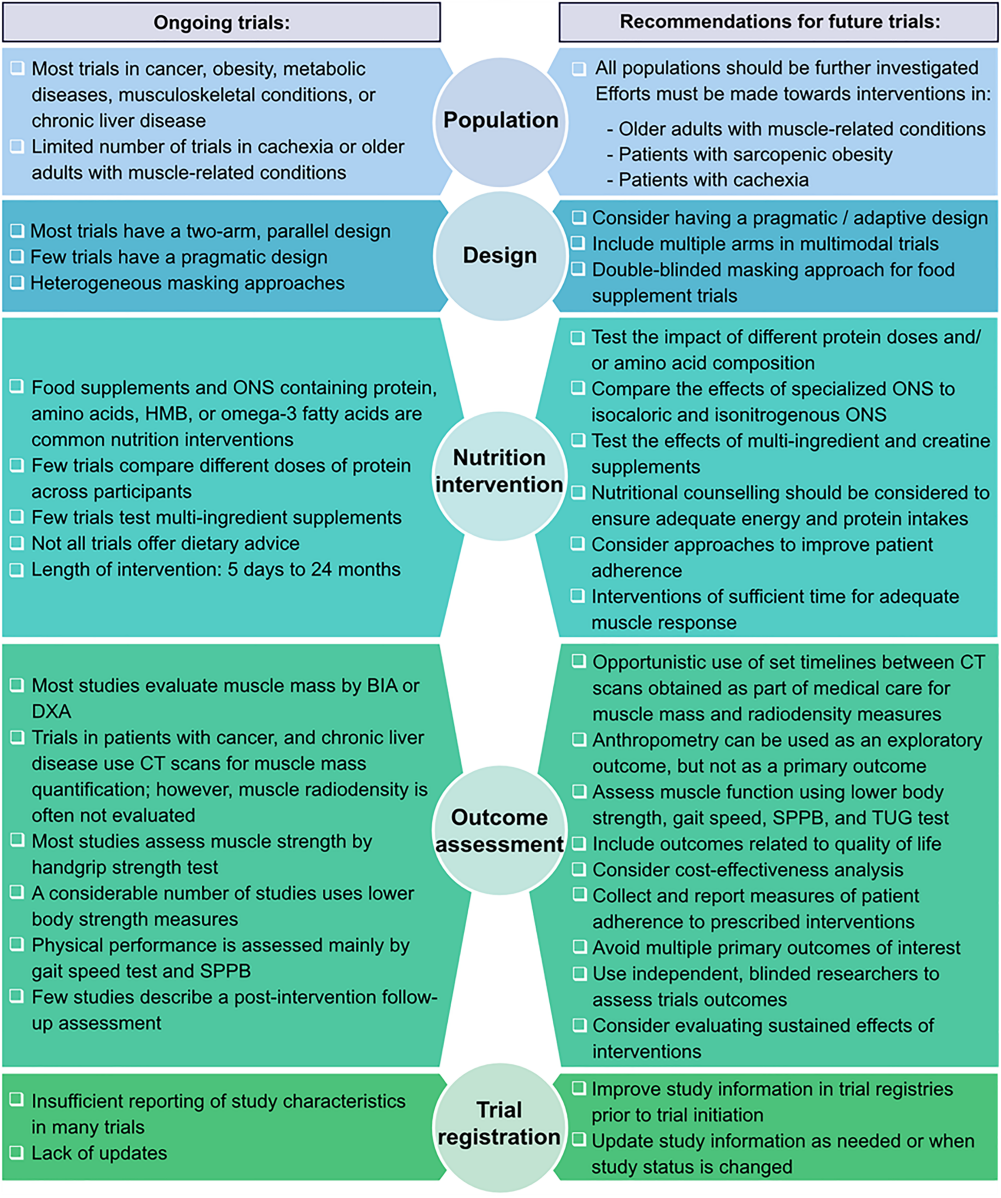
Recommendations for future trials investigating nutrition or multimodal interventions to prevent or treat low muscle mass or function, sarcopenia, or cachexia. BIA, bioelectrical impedance analysis; CT, computed tomography; DXA, dual‐energy X‐ray absorptiometry; ONS, oral nutritional supplement; SPPB, Short Physical Performance Battery; TUG, timed up and go.

In conclusion, this scoping review documented several ongoing RCTs exploring the role of nutrition interventions on muscle mass, muscle function, sarcopenia, or cachexia in aging and disease. We should expect additional evidence on the effects of food supplements and ONS containing protein, amino acids, omega‐3 fatty acids, or HMB on these conditions. Although many trials share similar types of interventions, methodological heterogeneity was observed regarding study design, supplement dosage, length of intervention, and outcome assessment, and essential information was missing for some trials. These issues/limitations may hinder comparisons between studies, data‐pooling, and future meta‐analyses, which in turn limits the potential of these ongoing studies to inform evidence‐based recommendations. To advance the field, here, we provide a set of recommendations for future trials on nutrition and multimodal interventions addressing muscle health, sarcopenia, and cachexia (*Figure* 7; a table version of this is included in *Table* S8).

## Conflict of interest

C.E.O. has received honoraria from Abbott Nutrition outside the scope of the submitted work. C.M.P. has received honoraria and/or paid consultancy from Abbott Nutrition, Nutricia, Nestlé Health Science, Fresenius Kabi, Pfizer, and Helsinn. A.L. has received honoraria and/or paid consultancy from Abbott, Baxter, BBraun, Fresenius Kabi, Nestlé Health Science, Nutricia, and Smartfish; and research grant from Fresenius Kabi. The other authors declare that they have no known conflicts of interest.

## Supporting information


**Figure S1.** PRISMA Flow Diagram for Systematic Reviews of Databases and Registers [42, 43]. Note one included trial reported two separate studies, hence *N* = 113 included trials. Abbreviations: NE, not eligible; WHO ICTRP, World Health Organization International Clinical Trials Registry Platform.

**Table S1.** Search terms.
**Table S2** – Inclusion and exclusion criteria according to PICOS (population, intervention, comparator, study design) and other relevant information.
**Table S3** – Trials excluded due to lack of information on the type of nutrition intervention.
**Table S4** – Data extraction spreadsheet.
**Table S5** – Details regarding dietary advice interventions in clinical and non‐clinical conditions.
**Table S6** – Details regarding food supplements and oral nutritional supplements (ONS) in clinical conditions.
**Table S7** – Details regarding food supplements and oral nutritional supplements (ONS) in non‐clinical conditions.
**Table S8** – Recommendations for future trials investigating nutrition or multimodal interventions to prevent or treat low muscle mass or function, sarcopenia, or cachexia.
