# Supplementary material for: Mapping ongoing nutrition intervention trials in muscle, sarcopenia, and cachexia: a scoping review of future research
Source: J Cachexia Sarcopenia Muscle. 2022 Mar 17;13(3):1442–59. doi: 10.1002/jcsm.12954 (PMC9178172; doi:10.1002/jcsm.12954)
Supplement: Supplementary file 1 — Figure S1. PRISMA Flow Diagram for Systematic Reviews of Databases and Registers [42, 43]. Note one included trial reported two separate studies, hence N = 113 included trials. Abbreviations: NE, not eligible; WHO ICTRP, World Health Organization International Clinical Trials Registry Platform. [file JCSM-13-1442-s002.pdf]

Supporting information

Mapping ongoing nutrition intervention trials in muscle, sarcopenia, and cachexia: a scoping review of future research

Journal of Cachexia, Sarcopenia, and Muscle

Camila E. Orsso[1], Montserrat Montes-Ibarra[1], Merran Findlay[2], Barbara S van der Meij[3,4,5,6], Marian de van der Schueren[5,6], Francesco Landi[7,8], Alessandro Laviano[9], Carla M. Prado[1]*

[1] Human Nutrition Research Unit, Department of Agricultural, Food and Nutritional Science, University of Alberta, AB, Canada.
[2] Cancer Services, Royal Prince Alfred Hospital, Camperdown, NSW, Australia.
[3] Bond University Nutrition and Dietetics Research Group, Faculty of Health Sciences and Medicine, Bond University, Gold Coast, Queensland, Australia.
[4] Department of Dietetics and Foodservices, Mater Health Services, Mater Hospital, South Brisbane, Queensland, Australia.
[5] Department of Nutrition, Dietetics and Lifestyle, School of Allied Health, HAN University of Applied Sciences, Nijmegen, the Netherlands.
[6] Department of Human Nutrition and Health, Wageningen University and Research, Wageningen, the Netherlands.
[7] Department of Geriatrics, Neurosciences and Orthopaedics, Catholic University of the Sacred Heart, Rome, Italy.
[8] Geriatric Department, Fondazione Policlinico Universitario "Agostino Gemelli" IRCCS, Rome, Italy.
[9] Department of Translational and Precision Medicine, Sapienza University of Rome, Rome, Italy.

Corresponding author
Professor Carla M. Prado, PhD, RD
2-021 Li Ka Shing Centre for Health Innovation
University of Alberta
Edmonton, AB, Canada T6G 2E1
Tel: 780.492.7934 / Fax: 780.492.9555

Supporting information

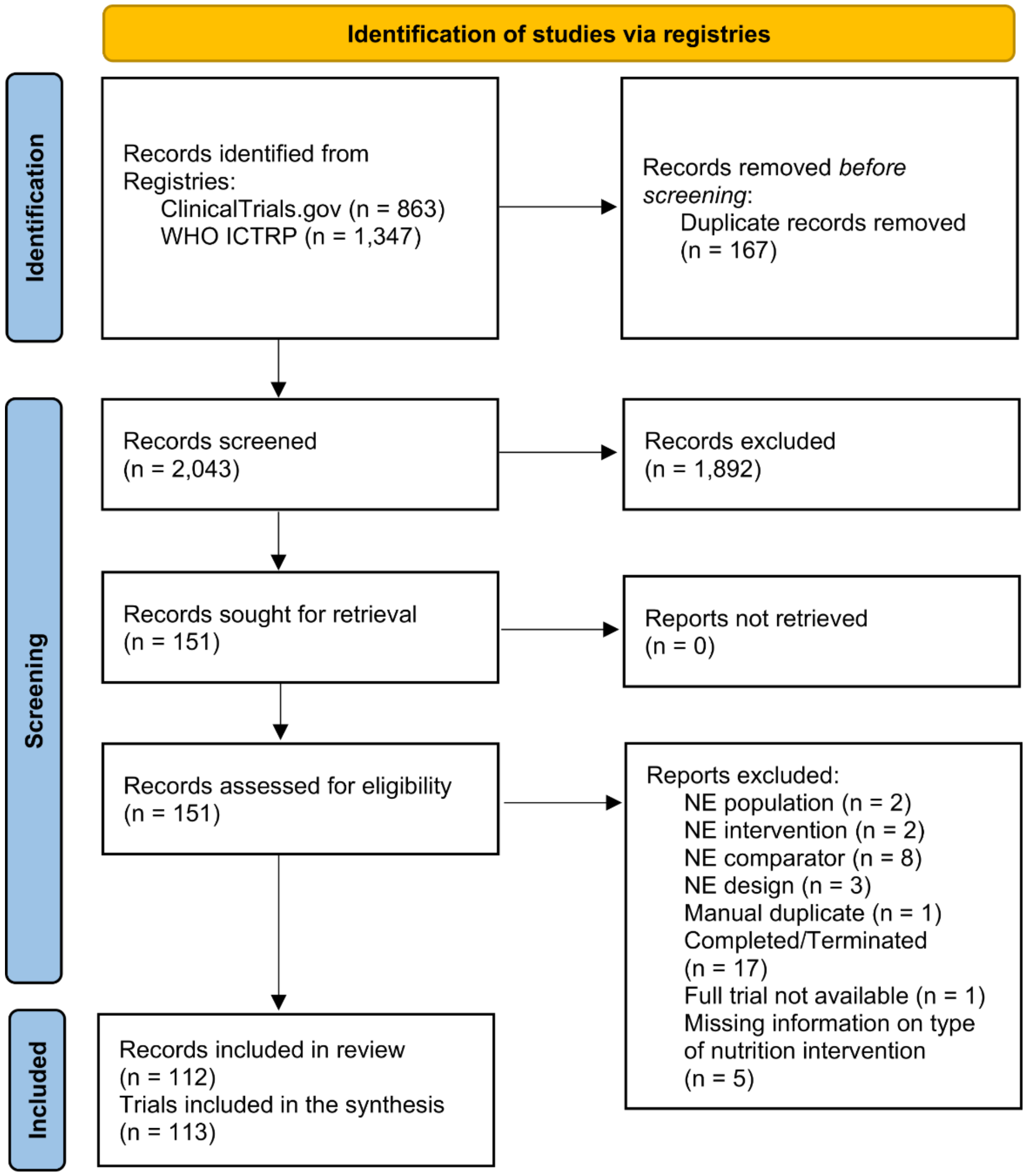

Figure S1 – PRISMA Flow Diagram for Systematic Reviews of Databases and Registers [42,43]. Note one included trial reported two separate studies, hence N = 113 included trials. Abbreviations: NE, not eligible; WHO ICTRP, World Health Organization International Clinical Trials Registry Platform.
